# Characterization of thymine microcrystals by CARS and SHG microscopy

**DOI:** 10.1038/s41598-020-74305-4

**Published:** 2020-10-13

**Authors:** Andrej Dementjev, Danielis Rutkauskas, Ivan Polovy, Mindaugas Macernis, Darius Abramavicius, Leonas Valkunas, Galina Dovbeshko

**Affiliations:** 1 Center for Physical Sciences and Technology, Sauletekio Ave. 3, 10257 Vilnius, Lithuania; 2 Institute of Physics, National Academy of Sciences of Ukraine, 46 Nauki Ave, Kyiv, 03680 Ukraine; 3 Institute of Chemical Physics, Vilnius University, Sauletekio Av. 9, 10222 Vilnius, Lithuania

**Keywords:** Materials science, Mathematics and computing, Optics and photonics

## Abstract

Identification of chemically homologous microcrystals in a polycrystal sample is a big challenge and requires developing specific highly sensitive tools. Second harmonic (SHG) and coherent anti-Stokes Raman scattering (CARS) spectroscopy can be used to reveal arrangement of thymine molecules, one of the DNA bases, in microcrystalline sample. Strong dependence of CARS and SHG intensity on the orientation of the linear polarization of the excitation light allows to obtain high resolution images of thymine microcrystals by additionally utilizing the scanning microscopy technique. Experimental findings and theoretical interpretation of the results are compared. Presented experimental data together with quantum chemistry-based theoretical interpretation allowed us to determine the most probable organization of the thymine molecules.

## Introduction

The nucleic acid bases—thymine (5-methyluracil), adenine, cytosine, uracil and guanine through hydrogen bonds and stacking interactions self-assemble into nanostructures of different dimensionality^1,2^. While such structures are not found in Nature, detailed information on intermolecular forces which hold the DNA structure, becomes encoded in crystal forms, formation energies as well as in thermodynamic properties of the crystals^3–5^. The ordering of this self-arrangement depends on the type of substrate used for the assembly. For example, on the Cu(111) surface, thymine aggregates into small clusters, whereas other DNA bases form ordered one- and two-dimensional structures^6^. At the same time, on the single-wall carbon nanotubes, adenine forms mostly islands, while thymine self assembles into single or multilayer film structures^7^. In addition to different crystallization character of different bases, the computationally-guided search for different solid forms revealed that regular thymine structures also exhibit the crystal polymorphism, where it adopts more than one crystal form^8^ with very similar X-ray diffraction, IR, and Raman spectroscopic characteristics. The different polymorphs could be distinguished by employing a wide range of complementary analytical tools ranging from the hot-stage microscopy to the water activity measurements^8^. The detailed studies of the vibrational spectra of thymine, uracil and its derivatives have been performed using IR, FT-IR and Raman spectroscopies in combination with ab initio modeling^9–14^.

In the present work, we apply a combined experimental and theoretical approaches of the microscopic imaging of the second harmonic generation (SHG) and the coherent anti-Stokes Raman scattering together with the density functional theory (DFT) calculations to investigate polymorphism of the thymine crystals. CARS and SHG imaging technique has been used previously for determining bone and collagen morphologies^15^. Compared to the regular bulk spectroscopic methods, the optical imaging allows for the detection of the possible structural heterogeneity on the length scale of the optical resolution. Then, the combination of a few different imaging modalities^16^ permits the spatial mapping of the sample areas with different characteristics that would otherwise be averaged out in a bulk measurement.

The SHG is a second order non-linear optical process that occurs in the ordered materials with non-centrosymmetric structure and is thus sensitive to the macroscopic details of the self-assembly^17^. Because of this, the SHG imaging has been, for example, adopted as a label-free method to obtain the information on the detailed structural arrangement of the various biological macromolecules such as collagen in the extracellular matrix^18^.

The coherent version of the Raman scattering—CARS, features orders of magnitude higher signal rates compared to the regular Raman scattering^19^ and can therefore be employed for microscopic imaging with acceptable acquisition timings^20^. The CARS signal originates from the vibrational signature of the material that is connected to the structure of the chemical bonds. To identify the relationship between the structure and properties of the material, the effectiveness of orientational mapping using polarized Raman^21^ or CARS^22,23^ microscopy was demonstrated. While the techniques have long history in material research, the bulk nonlinear optical studies of thymine to date^24–27^ were not able to distinguish the different polymorphs of thymine crystals.

In this study, we performed the combined excitation polarization orientation-dependent CARS/SHG imaging of the glass-deposited thymine crystals. The quantitative dependences of the intensities of the non-linear signals on the orientation of the excitation polarization were related to the quantum chemical calculations. Experiments demonstrated several structural motifs even in the same crystalline droplets. Detailed analysis of self-assembly possibilities allowed us to conclude that the most probable structural unit of the measured thymine crystals was the thymine-thymine inverse or thymine–thymine symmetric dimer.

## Methods

### CARS system

**Figure 1:**
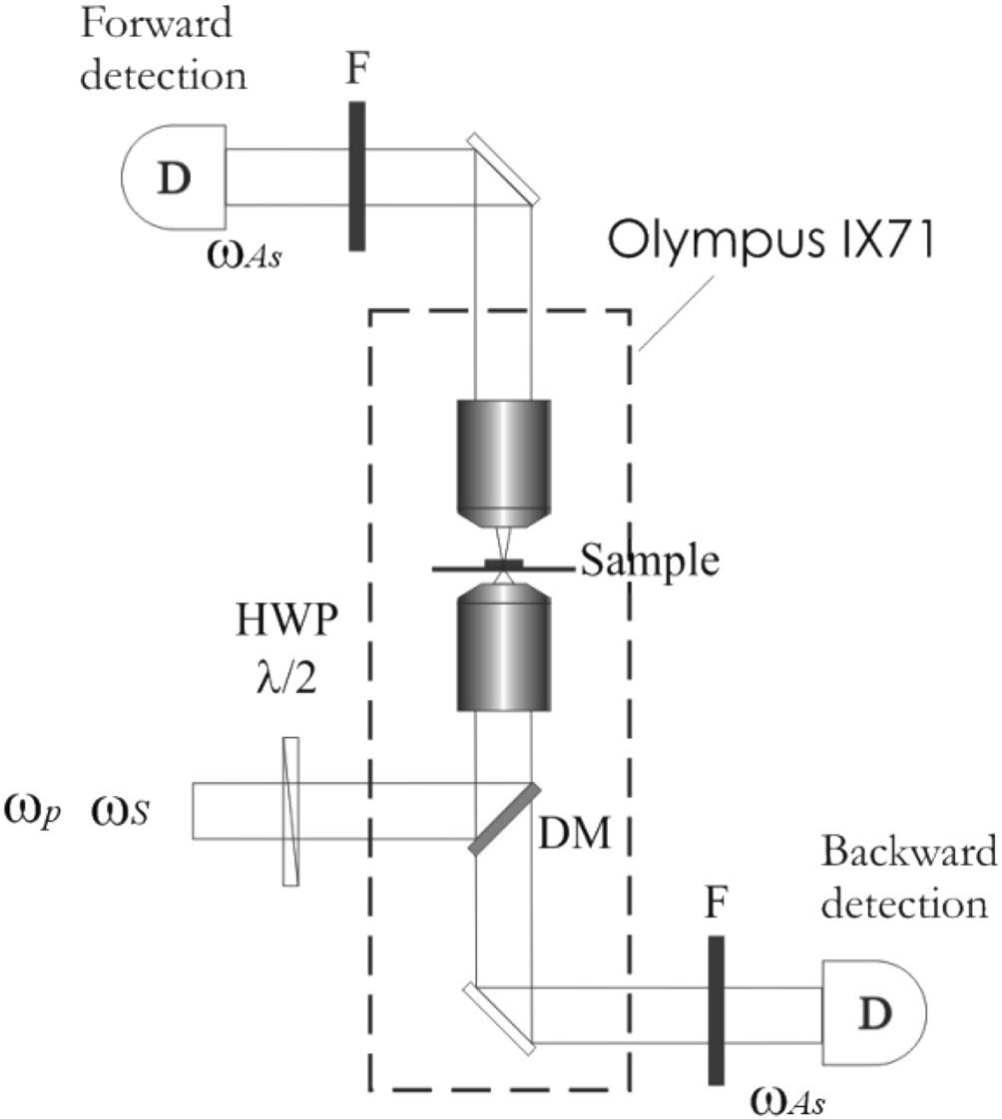
The CARS microscope setup. *HWP* achromatic half wave plate, *DM* dichroic mirror, *F* filters.

The microscopic imaging was performed using the custom-built CARS system (Fig. 1)^23^. Commercial Olympus IX71 microscope in combination with the dual-wavelength 1 MHz picosecond laser source (Ekspla) and the piezo scanning system P-517.3CL (Physik Instrumente) was utilized for the raster-scanning of the sample. The excitation light was reflected off the custom-made (Optida) dichroic mirror and focused onto the sample with the oil-immersion objective Plan Apochromat, 60 × , NA 1.42 (Olympus). The CARS signal was detected with the avalanche photodiode module SPCM-AQRH-14 (Perkin Elmer), connected to the multifunctional PCI board 7833R (National Instruments) either in the forward or backward direction. The fundamental wavelength (1064 nm) and tunable-wavelength light from the optical parametric generator (OPG) were used as “Stokes” (*ω*_S_) and “Pump” (*ω*_p_) excitation beams, respectively. The fingerprint region was studied in the range from 1250–1700 cm^-1^. For this, the OPG was tuned from 938 to 900 nm and the resulting CARS signal (*ω*_AS_ = 2*ω*_p_ − *ω*_S_) from 840 to 782 nm was detected. Long-pass (cut-off at 860 nm) and short-pass (cut-off at 780 nm) filters were applied to spectrally separate the CARS signal in the forward-detection scheme. To separate the second harmonic generated using the fundamental *ω*_S_ (1064 nm) beam, the bandpass filter FLH532-10 (Thorlabs) centered at 532 nm with FWHM of 10 nm, was used^24^. The excitation powers of 1 mW and 3 mW were used for the “Pump” and “Stokes” beams, respectively.The direction of the linear polarization of the incoming light was controlled by the achromatic half-wave plate AHWP05M-980 (Thorlabs). To provide a linearly polarized light we used a metallic mirror instead of a dichroic mirror (DM in Fig. 1) in order to avoid elliptical polarization as well as polarization-dependent transmission by conventionally used dielectric DM. The modified optical setup was tested by using linear polarizer placed after the excitation objective and measuring the intensity of linearly polarized light for polarization range from 0° to 180°.

### Thymine sample

The thymine crystals were prepared by depositing and drying 20 µL of 1 mg/mL solution of thymine^28^ (T0376, Sigma-Aldrich) in ultra-pure water (Labostar, Siemens) on an untreated glass coverslip (Menzel Glaser, Braunschweig, Germany). The sample preparation was carried out under ambient conditions: at room temperature and relative humidity of about 40%. The results of the sample preparation procedure were well reproducible.

### Computations

The quantum chemistry calculations were performed for the thymine single molecules and for various dimers. After the primary analyses the two dimer configurations were chosen as final structures due to the fact that they allow a straightforward repetition of the structure to form the periodic crystal structure. The DFT calculations were carried out for the structure optimization, the Raman spectra, and the dipole moments of thymine monomer and dimers using the Gaussian package^29^. The Raman spectra were obtained with the B3LYP functional with the LANL2DZ basis sets, which is sufficiently accurate in the case of the thymine molecule^30^. The method was used for the ground and the first excited state dipole moment calculations. The dipole moment calculations were repeated with B3LYP/DGDZVP, which is reported to be more precise for the purpose^31^. In order to check the importance of the dispersion correction in Raman calculations^32–34^ we recalculated the final results at APFD/aug-cc-pVDZ calculation level. For the calculations of this type the computational walltime increased by 5 times. However, the results did not change considerably (the scaling factor was required also) and for this reason the calculation level used in this paper was as it was proposed previously in the literature^30,31^. BSSE values of both final thymine dimers were ≈0.12 eV, ≈0.05 eV and 0.04 eV for B3LYP/LANL2DZ, B3LYP/DGDZVP and APFD/aug-cc-pVDZ, respectively.

## Results

**Figure 2:**
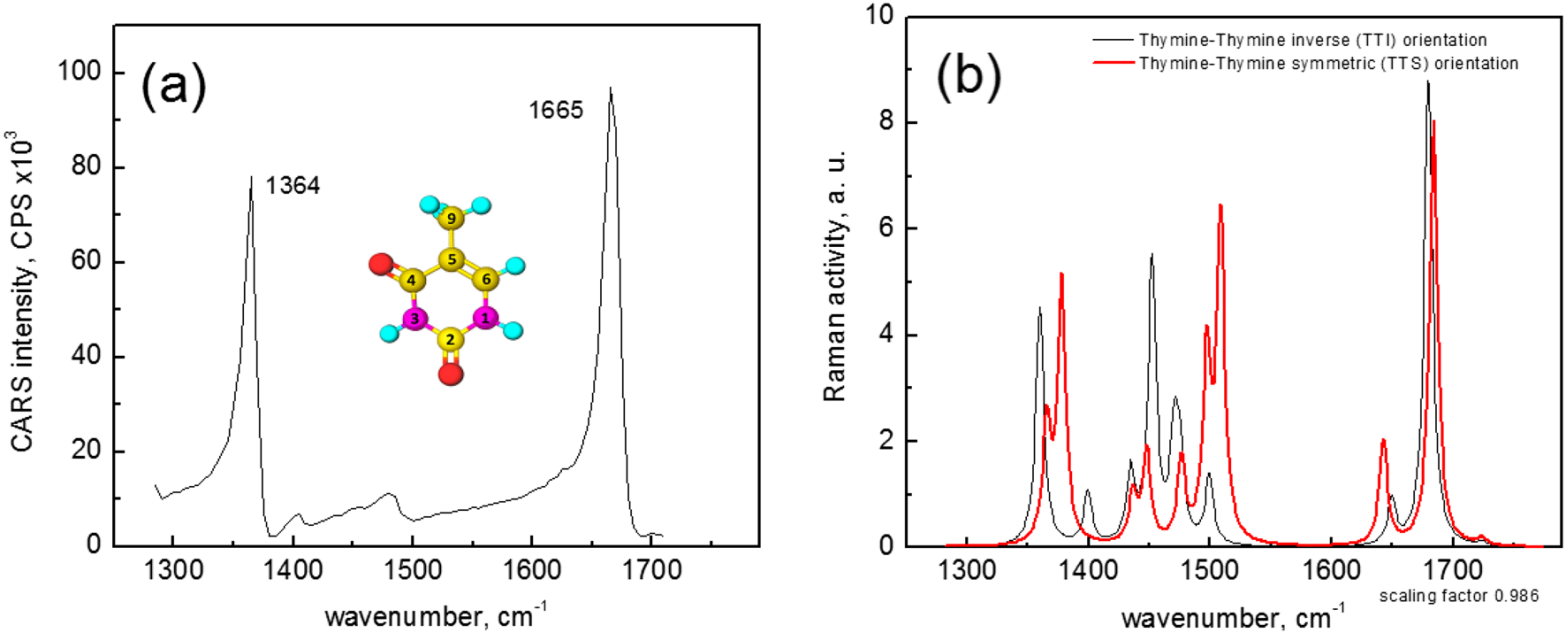
The experimental CARS spectrum **(a)** of thymine microcrystal contains intense bands at 1364 and 1665 cm^−1^ assigned to δN(3)-H plus δs C–H_3_ bending as well as ν C(4)=O stretching vibration, respectively. Insert: the structure of thymine molecule. Calculated Raman spectrum of the thymine dimers is depicted in **(b)**.

Thymine preparations on a glass coverslip were probed by a combination of the CARS and SHG microscopies. The CARS, being a chemically selective method, allowed us to identify the thymine crystals. The CARS spectrum of the studied sample is presented in Fig. 2a. Most intense bands at 1364 and 1665 cm^−1^ were assigned to δN(3)-H plus δs C–H_3_ bending as well as ν C(4) = O stretching vibration of thymine molecule, as suggested by theoretical calculations (vide infra). Less noticeable spectral features at 1405 and 1480 cm^−1^ should be assigned to stretch νC(2)N(3) and bend δN(1)H vibrations, respectively. These resonance bands have a typical asymmetrical CARS profile indicating the coherent mixing of resonant contribution with the non-resonant background ^35^. Such a contribution is insignificant for the 1364 and 1665 cm^−1^ bands, enabling the chemically selective imaging of thymine with high vibrational contrast.

The calculated Raman spectrum of thymine monomer in the same spectral range is presented in Fig. 2b. The presented spectrum is the result of the raw ab initio calculation obtained without any arbitrary rescaling. The similarity of the band patterns of the calculated (Table 1) and the measured spectra allowed us to assign the experimental spectral peaks unambiguously. These results are corroborated also by the previous study^10^.

**Table 1:** Thymine vibrational bands in the fingerprint region.

Monomer	TTI dimer	TTS dimer	Assignments
1711 vs	1703 vs	1708vs	StrC5=C6, Def R(strC4-C5), StrC4**=**O, A bend N1-H, C6-H
1484 m	1473w 1491vw 1496vw	1530 m	S bend N1-H, C6-H, Sciss C9-H10, C5
1454 w	1454 vw	1457vw	Umb CH3
1382 vs	1379vs	1397vs 1385w	S bend N1-H, C6-H

*s* strong, *vs* very strong, *m* medium, *w* weak, *vw* very weak.

The main features of the experimental Raman spectrum of the solid thymine^30^ are reproduced by the calculated Raman activity of the thymine-thymine inverse (TTI) orientation dimer with the scaling factor of 0.986 (Table 1, Fig. 2b).

**Figure 3:**
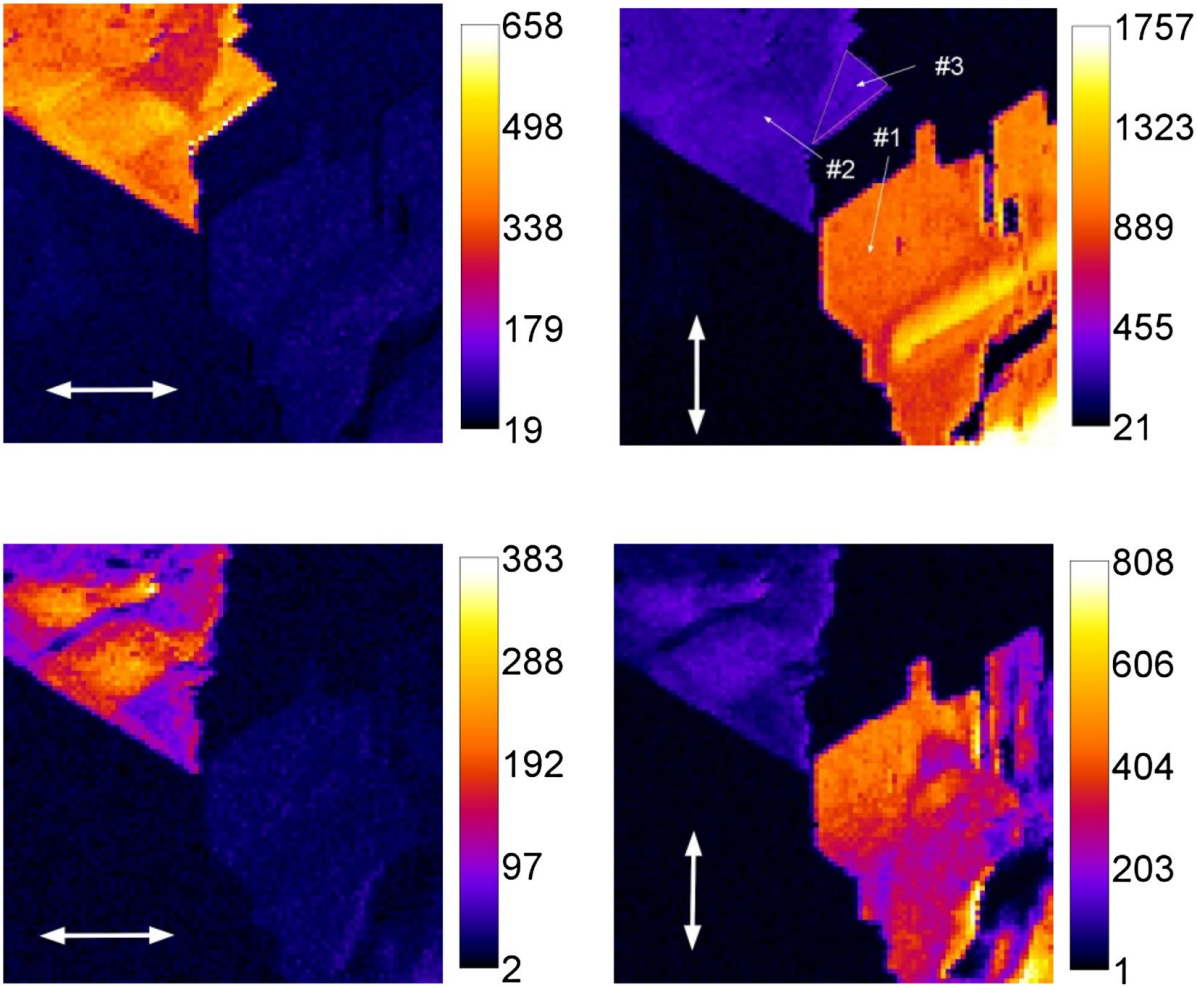
The CARS (top) and SHG (bottom) images of thymine with two perpendicular linear polarizations of the incoming light indicated with white arrows. The areas #1 and #2 appear in the image at different polarization orientations of the incoming light. The region #3 is noteworthy for its lack of the SHG. The size of the images is 20 × 20 µm. CARS and SHG images obtained at the same height of the sample. Lateral resolution was ~ 0.9 µm.

The microscopic CARS image recorded at the fixed 1665 cm^−1^ wavenumber exhibits straight border crystalline structures (Fig. 3), which could also be observed in the transmitted light. In addition a strong monochrome emission from a sample was detected, and related to the second harmonic from the incoming radiation. The SHG signal was detected both from the “Pump” and the “Stokes” irradiation. Moreover with the “Stokes” beam intensity of several mW, the SHG could be observed visually in the transmission mode. Because of the higher sensitivity of the detector at 532 nm, for the excitation in the SHG experiment we employed the “Stokes” beam (1064 nm). In order to evaluate the SHG efficiency, we compared the intensity of the SHG signal from the thymine with that from the Z-cut quartz-plate (POQB-168462, Precision Micro-Optics). With the tight focusing in the microscope, the second harmonic is generated at the length scale shorter than the sample thickness. The phase-matching condition is therefore fulfilled automatically and the intensity of the response does not depend on the thickness of the sample and is characteristic of the material. We found that at 1 mW of incoming first harmonic (1064 nm) the intensity of the SHG signals were 850 and 960 counts for quartz-plate and thymine, respectively, indicating a similar efficiency of SHG in these materials.

The intensity of both, CARS and SHG signal of thymine strongly depends on the orientation of the linear polarization of the excitation light (the polarization of the emitted light was not considered). Figure 3 demonstrates images for two mutually orthogonal orientations of the linear polarization of the incoming light. It is evident that two adjacent objects in the image feature maximum brightness at different orientations of the polarization of the incident light.

**Figure 4:**
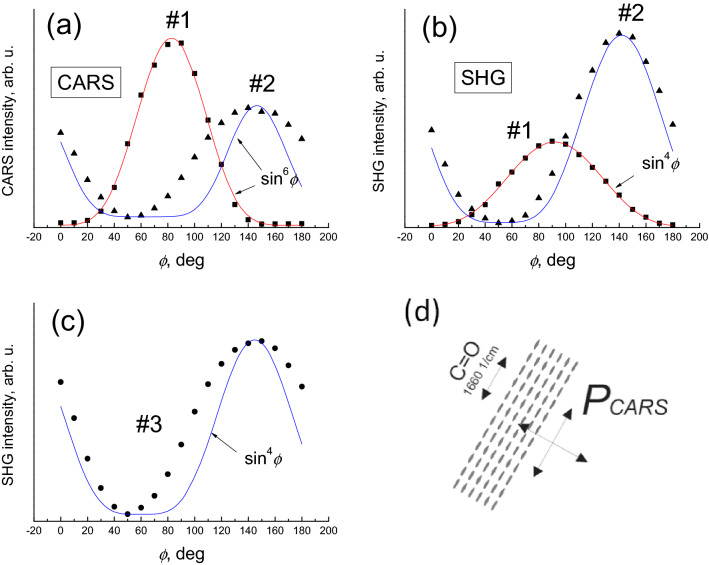
The CARS intensity dependence on the direction of the linear polarization of the incoming light **(a)**. The same angular dependence for the SHG **(b)**. The experimental points are presented for the areas designated in Fig. 3 as #1 (squares) and #2 (triangles). The area denoted in Fig. 3 as #3 does not exhibit SHG and thus only its angular dependence of the CARS intensity is presented **(c)**. The polarization *P*_*CARS*_ induced in a crystal of thymine depends on the orientation of the incident light polarization relative to the orientation of the Raman oscillators in the crystal **(d)**.

Recently, the suitability of the CARS approach to determine the molecular orientations was demonstrated by analyzing the angular dependencies of the CARS intensity in the liquid crystals ^22^. Theoretical considerations^23^ predict the sin^8^*ϕ* dependence of the CARS intensity on the angle between the molecular transition dipole moment and the polarization orientation of the laser field. Since our detection was not emission polarization-sensitive, the angular dependence of the CARS intensity should be reduced to sin^6^*ϕ*^36^. The dependence of the CARS/SHG intensity on the polarization direction of the excitation beam is presented in Fig. 4.

Each point in the plot of the angular dependence of the CARS and SHG intensity is the average over the same region (~ 2 µm × 2 µm) in the crystal. Experimental data is presented for two different objects (crystals) denoted in Fig. 3 as #1 and #2. The angular positions of the maximum signal for these two crystals are shifted approximately by 60 degrees for both the CARS and SHG indicating different orientation of the crystal lattice. The maximum amplitude of the CARS in crystal #1 is greater than in crystal #2, and vice versa for SHG.

The CARS data for the crystal #1 was closely approximated with the sin^6^*ϕ* dependence. The minimum of the CARS signal at *ϕ* = 0 (or 180) indicates that the polarization of the sample created by the driving field approaches zero for those orientations of the irradiation polarization.

In turn, SHG data for the crystal #1 shows sin^4^*ϕ* dependence. To characterize the SHG angle dependence we resorted to the nonlinear response function theory ^37^. In the second order approximation the induced polarization can be expressed as

$${P}_{\gamma }^{\left(2\right)}\left(2\omega \right)={\sum }_{\beta \alpha }{\chi }_{\gamma \beta \alpha }^{\left(SHG\right)}{E}_{\beta }\left(\omega \right){E}_{\alpha }(\omega )$$

here, ${E}_{\alpha }(\omega )$ is the amplitude of the projection of the $\alpha$ orientation of the optical field at frequency $\omega$, ${P}_{\gamma }^{\left(2\right)}\left(2\omega \right)$ is the amplitude of the induced polarization at the double frequency, ${\chi }_{\gamma \beta \alpha }^{\left(SHG\right)}$ is the susceptibility tensor. In the case of off-resonant conditions, the leading term in the susceptibility tensor can be written as

$${\chi }_{\gamma \beta \alpha }^{\left(SHG\right)}\propto {\Pi }_{\beta \alpha }\left({\mu }_{ge}\cdot \gamma \right)\left({\mu }_{ee/gg}\cdot \beta \right) \left({\mu }_{eg}\cdot \alpha \right).$$

Here, ${\Pi }_{\beta \alpha }F\left(\beta ,\alpha \right)=F\left(\beta ,\alpha \right)+F\left(\alpha ,\beta \right)$ is the permutation operator over the incident field polarizations, ${\mu }_{eg}$ is the electronic transition dipole, while ${\mu }_{ee/gg}={\mu }_{ee}-{\mu }_{gg}$ is the permanent difference dipole. Assuming that incident fields have the same polarization (e.g. $\alpha$), while the detection is polarization-insensitive, we have ${\chi }_{\gamma \alpha \alpha }^{\left(SHG\right)}\propto \left({\mu }_{ge}\cdot \gamma \right)\left({\mu }_{ee/gg}\cdot \alpha \right)\left({\mu }_{eg}\cdot \alpha \right)$. If we consider a single excited state of the material, in general, the transition dipole and permanent dipole could have different orientations. We can then introduce the angular dependencies, $\left({\mu }_{ee/gg}\cdot \alpha \right)=\left|{\mu }_{ee/gg}\right|\mathrm{cos}\psi$ and $\left({\mu }_{eg}\cdot \alpha \right)=\left|{\mu }_{eg}\right|\mathrm{cos}\phi$ and obtain

$${\chi }_{\alpha \alpha \alpha }^{\left(SHG\right)}\propto \left({\mu }_{ge}\cdot \gamma \right)\left|{\mu }_{eg}\right|\left|{\mu }_{ee/gg}\right|\left[{\mathrm{cos}}^{2}\phi \mathrm{cos}\delta -\frac{1}{2}\mathrm{sin}2\phi \mathrm{sin}\delta \right].$$

Here, we denoted $\delta =\psi -\phi$. Considering the power of the field to be proportional to ${\left|{P}_{\gamma }^{\left(2\right)}\left(2\omega \right)\right|}^{2}$, from the first term of the equation we obtain the ${\mathrm{cos}}^{4}\phi$ dependence, while the second term would introduce additional modulation. The experiment in Fig. 4 exhibits the ${\mathrm{cos}}^{4}\phi$ dependence without the additional modulation, consequently we find that $\delta =0$, i. e., the transition and permanent dipoles have the same orientation.

The polarization dependences for region # 2 deviate strongly from the expected regularities sin^6^*ϕ* (for the CARS) and sin^4^*ϕ* (for the SHG). We attribute this to the inhomogeneity of the crystal structure. As mentioned earlier, to improve the signal-to-noise ratio, each point in the plots of Fig. 4 is the result of averaging the intensity over the area—in this case, 2 by 2 microns. Similar heterogeneity is also characteristic for region # 3. On the other hand, the polarization curves tightly following the expected dependences for region #1 indicating crystal homogeneity over that area.

**Figure 5:**
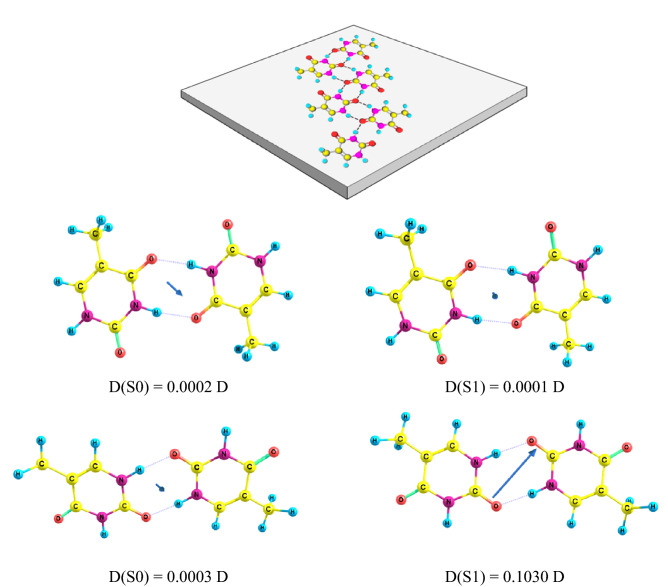
Possible arrangement of the thymine molecules in the crystal in the plane of the coverslip is shown at the top. Below are shown two optimized structures of thymine dimers (DFT computations), which could be extended into periodic crystal organization. Permanent dipoles of the ground state (D(S0)) and the lowest energy electronic excited state (D(S1)) are given as well.

The electronic transitions are responsible for both, the CARS and SHG. It is thus natural to assume that the same electronic excitation is involved in both signals. Obtained polarization dependence of either CARS or SHG is, evidently, determined by orientation of thymine molecule. The thymine molecule is flat, and its polarization can be induced only in the plane of the molecule. In the experiment, the laser polarization was rotated in the plane of the surface of the coverslip, and the maximum/minimum alteration of the intensity of CARS or/and SHG signal can correspond to either parallel or perpendicular orientation of the molecular plane with respect to the plane of the surface of coverslip (see Fig. 5 top). In the absence of the chemical bonding with the substrate it is natural to assume that the molecules lie flat on the substrate and their electronic dipoles are in the plane of the coverslip (Fig. 5 top). Further, because the thymine favors form a cluster structures based on both hydrogen bonding and stacking interactions^14^, it is reasonable to assume that for crystal the orientation of molecules in parallel to the plane of the coverslip is also valid. Consequently, all the molecules in the crystal show a long-range ordering parallel to the surface of the coverslip as shown in Fig. 5.

SHG generation requires large permanent dipoles so a single structural unit of the crystal (a unit cell) must correspond to a simple structure, of course, utilizing hydrogen bonding pattern of thymine molecules. We thus find that thymine dimeric structures allow for such structure formation. Figure 5 shows optimized thymine dimer configurations obtained by quantum chemistry simulations where both molecules are in the planar configuration. For the final study in this paper we select two types of dimers. Thymine-thymine inverse (TTI) orientation and thymine-thymine symmetric (TTS) orientation (Fig. 5 bottom). The TTI and TTS configurations allow a straightforward repetition of the structure to form the periodic crystal structure. The calculations of the alternative dimer configurations resulted in the out-of-plane deformations or the lack of defined hydrogen bond pattern for the long periodic structures. Some of the dimers had the dipole moments up to 20 D in the excited state but in repeated dimer structure the dipole moment summed to zero. Consequently, TTI and TTS configurations can be considered as possible candidates for the thymine crystallization. The TTI and TTS ground state dipole moments should be considered as equal to zero. The excited state dipole moment of the TTS was 0.1 D (Fig. 5) while for TTI it was zero. Repetition of the molecular dipoles yields molecular ribbons that form the microscopic crystals. So TTS is the best candidate to characterize the SHG response.

In the images presented in Fig. 3 the area #3 exhibits intense CARS and no SHG. Moreover, the CARS intensity and the angular dependence in this region are similar to the ones obtained for the areas #1 and #2. Consequently, the crystal ordering of the molecules is maintained in area #3. However, the CARS is generated by the four dipole-field interactions so it is generated by the parallel and anti-parallel dipole configurations. At the same time, the SHG is generated by the three system-field interactions, and can occur only in the case of all microscopic dipoles being strictly parallel on macroscopic scale. From this it follows that in the areas #1 and #2 all molecular ribbons in the crystal are oriented parallel and point in the same direction. At the same time, the area #3 is formed by anti-parallel ribbon configuration.

## Discussion and conclusions

Recorded images and polarization dependences of CARS/SHG describe microscopic properties of crystalline thymine. Thymine packaging arrangement has been proposed by Braun et al.^8^. Packaging starts from hydrogen bonded ribbons, which can be differently arranged within layers that stack into thymine crystal. According to Braun et al. there are four types of ribbons which differ in the structure of their dimers. Combinations of ribbons in crystal layer give, in turn, four types of packing arrangements noted as *Anhydrate A, Anhydrate B, Anhydrate C, Anhydrate D*^8^.

If these four configurations are considered in terms of the orientation, then for the molecules oriented differently either in the ribbons or in the layers, the CARS response would not depend on the direction of the polarization of the excitation. This is not the case in our experiment. Thus it should be concluded that the thymine packing corresponds to the mode denoted in^8^ as *Anhydrate C*. Only this anhydrate contains the Raman oscillators (Fig. 2a) oriented in the same direction. Any other packing modes of the thymine molecules, denoted in^8^ as *Anhydrate A, Anhydrate B, Anhydrate D,* have at least two different orientations of the Raman oscillator and therefore should exhibit the angular modulation different from that shown in Fig. 5. Based on this, we can conclude that for the case when the thymine molecules lie in the plane of the substrate, the packing is as of *Anhydrate C*. In our notation, the TTI and TTS are the most probable configurations to form the thymine crystals by self-organization.

Combined SHG and Raman microscopies have been used to reveal structures of thymine crystals in this paper and this constitutes a unique approach for molecular crystal characterization. This is a step ahead compared to a single Raman microscopy techniques^21,38,39^. The Raman signal does not require macroscopic ordering, consequently, the generated signal from a crystalline sample will necessarily contain a background from noncrystalline phase. The same holds in fluorescence-detected two-dimensional electronic microscopy, which has been used in characterization of photosynthetic molecular complexes^40^. The situation is completely different in SHG where the signal generation requires macroscopic ordering. So the signal when applied to crystal studies is background free. This has been efficiently employed in mapping e. g. biological samples^41,42^. By combining SHG and CARS modalities we get a high resolution information on local (microscopic) and global (macroscopic) picture of thymine ribbons. In this respect our approach is comparable to multiphoton multimodal generation microscopy^43^, which has been used to map various structures of e. g. atomically thin tungsten diselenide^44^.

The presented experiment demonstrates perfect self-assembly of thymine molecules into two crystal configurations. X-ray diffraction imaging, scanning tunneling microscopy, and others techniques can be used to recognize which configuration is realized in a single crystal detection. Imaging of several crystals in the same sample is out of reach by X-ray. In this work, we have shown that the method of optical nonlinear microscopy can be used when the sample contains several crystal homologues. Notice that generation of CARS and second harmonic are very efficient in thymine crystal and can therefore be used to detect tiny amounts of the sample. However, separate analysis of CARS or SHG data is not sufficient for unambiguous interpretation of crystal configuration: it can only be revealed by the combination of the CARS and SHG supported by quantum chemical calculations. Theoretical calculations of the Raman spectra and the dipole moment help to relate the CARS and SHG data with TTI and TTS configurations of the thymine crystallization. Unlike, for example, the X-ray diffraction imaging, the applied method of scanning microscopy provides an image of thymine crystals with high spatial resolution and gives information not only on the spatial distribution by the type of molecular packing in the crystal within several tens microns, but also yields crystal boundaries between different structural motifs. The scope of this article was limited to the description of the principles of such technology, leaving for the future detailed image processing allowing the formation of a two-color image of crystals highlighting the TTI and TTS regions with different colors.
